# Predictors of Care Home Admission and Survival Rate in Patients With Syndromes Associated With Frontotemporal Lobar Degeneration in Europe

**DOI:** 10.1212/WNL.0000000000209793

**Published:** 2024-08-30

**Authors:** Barbara Borroni, Barbara Tarantino, Caroline Graff, Johanna Krüger, Albert C. Ludolph, Fermin Moreno, Markus Otto, James B. Rowe, Harro Seelaar, Eino Solje, Elka Stefanova, Latchezar D. Traykov, Vesna Jelic, Sarah Anderl-Straub, Anne M. Portaankorva, Myriam Barandiaran, Alazne Gabilondo, Alexander G. Murley, Timothy Rittman, Emma Van Der Ende, John C. Van Swieten, Päivi Hartikainen, Gorana Mandić Stojmenović, Shima Mehrabian, Roberta Ghidoni, Antonella C. Alberici, Maria Teresa Dell'Abate, Chiara Zecca, Mario Grassi, Giancarlo Logroscino, for FRONTIERS

**Affiliations:** From the Department of Clinical and Experimental Sciences (B.B.), University of Brescia; Department of Continuity of Care and Frailty (B.B., A.C.A.), ASST Spedali Civili, Brescia; Medical and Genomic Statistics Unit (B.T., M.G.), Department of Brain and Behavioural Sciences, University of Pavia, Italy; Division of Neurogeriatrics (C.G.), Department NVS, Karolinska Institutet, Solna; Unit for Hereditary Dementia (C.G.), Theme Inflammation and Aging, Karolinska University Hospital-Solna, Stockholm, Sweden; Research Unit of Clinical Medicine (J.K., S.A.-S., A.M.P.), Neurology, University of Oulu; MRC (J.K., A.M.P.), Oulu University Hospital; Neurocenter (J.K.), Neurology, Oulu University Hospital, Finland; Department of Neurology (A.C.L., M.O.), University of Ulm; Deutsches Zentrum für Neurodegenerative Erkrankungen (DZNE) (A.C.L.), Ulm, Germany; Cognitive Disorders Unit (F.M., M.B., A.G.), Department of Neurology, Hospital Universitario Donostia; Neuroscience Area (F.M., M.B., A.G.), Biogipuzkoa Health Research Institute, San Sebastian, Spain; Department of Neurology (M.O.), Martin Luther University, University Hospital, Halle (Saale), Germany; MRC Cognition and Brain Sciences Unit (J.B.R., A.G.M., T.R.), Department of Clinical Neurosciences, and Cambridge University Hospitals NHS Trust, University of Cambridge, Cambridge, United Kingdom; Department of Neurology and Alzheimer Center Erasmus MC (H.S., E.V.D.E., J.C.V.S.), Erasmus MC University Medical Center, Rotterdam, the Netherlands; Neurology (E. Solje, P.H.), Institute of Clinical Medicine, University of Eastern Finland; Neurocenter (E. Solje), Neurology, Kuopio University Hospital, Finland; Neurology Clinic (E. Stefanova, G.M.S.), Faculty of Medicine, University Clinical Center, University of Belgrade; UH Alexandrovska (L.D.T., S.M.), Department of Neurology, Medical University Sofia, Bulgaria; Theme Inflammation and Aging (V.J.), Medical Unit Aging Brain, Karolinska University Hospital Huddinge, Solna; Division of Clinical Geriatrics (V.J.), Department NVS, Karolinska Institutet, Huddinge, Sweden; Molecular Markers Laboratory (R.G.), IRCCS Istituto Centro San Giovanni di Dio Fatebenefratelli, Brescia; and Center for Neurodegenerative Diseases and the Aging Brain (M.T.D., C.Z., G.L.), Pia Fondazione Cardinale Giovanni Panico, University of Bari-Aldo Moro, Italy.

## Abstract

**Background and Objectives:**

Data on care home admission and survival rates of patients with syndromes associated with frontotemporal lobar degeneration (FTLD) are limited. However, their estimation is essential to plan trials and assess the efficacy of intervention. Population-based registers provide unique samples for this estimate. The aim of this study was to assess care home admission rate, survival rate, and their predictors in incident patients with FTLD-associated syndromes from the European FRONTIERS register-based study.

**Methods:**

We conducted a prospective longitudinal multinational observational registry study, considering incident patients with FTLD-associated syndromes diagnosed between June 1, 2018, and May 31, 2019, and followed for up to 5 years till May 31, 2023. We enrolled patients fulfilling diagnosis of the behavioral variant frontotemporal dementia (bvFTD), primary progressive aphasia (PPA), progressive supranuclear palsy (PSP) or corticobasal syndrome (CBS), and FTD with motor neuron disease (FTD-MND). Kaplan-Meier analysis and Cox multivariable regression models were used to assess care home admission and survival rates. The survival probability score (SPS) was computed based on independent predictors of survivorship.

**Results:**

A total of 266 incident patients with FTLD were included (mean age ± SD = 66.7 ± 9.0; female = 41.4%). The median care home admission rate was 97 months (95% CIs 86–98) from disease onset and 57 months (95% CIs 56–58) from diagnosis. The median survival was 90 months (95% CIs 77–97) from disease onset and 49 months (95% CIs 44–58) from diagnosis. Survival from diagnosis was shorter in FTD-MND (hazard ratio [HR] 4.59, 95% CIs 2.49–8.76, *p* < 0.001) and PSP/CBS (HR 1.56, 95% CIs 1.01–2.42, *p* = 0.044) compared with bvFTD; no differences between PPA and bvFTD were found. The SPS proved high accuracy in predicting 1-year survival probability (area under the receiver operating characteristic curve = 0.789, 95% CIs 0.69–0.87), when defined by age, European area of residency, extrapyramidal symptoms, and MND at diagnosis.

**Discussion:**

In FTLD-associated syndromes, survival rates differ according to clinical features and geography. The SPS was able to predict prognosis at individual patient level with an accuracy of ∼80% and may help to improve patient stratification in clinical trials. Future confirmatory studies considering different populations are needed.

## Introduction

Frontotemporal lobar degeneration (FTLD) causes a heterogeneous group of neurodegenerative disorders with a wide range of clinical, genetic, and neuropathologic features.^1^ Behavioral and personality changes are among the most prominent symptoms in the behavioral variant frontotemporal dementia (bvFTD)^2^ while speech and language deficits are characteristic of primary progressive aphasia (PPA).^3^ A significant proportion of patients have associated extrapyramidal symptoms that may form part of either progressive supranuclear palsy (PSP)^4^ or corticobasal syndrome (CBS)^5^ while others present with overlapping motor neuron disease (MND), which defines the FTD-MND.^6^

In recent years, as the understanding of the molecular and clinical intricacies of FTLD has progressively improved, so too has the ability to develop novel interventions that might address the diverse and complex challenges posed by this disorder.^7,8^ However, treatment development, effective care, and social management remain difficult tasks, in part due to the extreme heterogeneity and the rareness of the disease. Knowledge of the natural disease history and predictors of disease trajectories is critical for ongoing efforts to assess the efficacy of treatment approaches and to promote adequate planning of public health service policies.

In this perspective, both care home admission rate, a pivotal event that predicts mortality, and survival rate may be considered reliable outcome measures of disease trajectories in dementia and in FTLD as well.^9^

No data on care home admission rates of patients with FTLD are yet available, and the available literature on FTLD survival is limited and often taken retrospectively from small clinical series, limited to selected phenotypes, without considering biological markers to exclude other neurodegenerative diseases such as Alzheimer disease, and applying earlier clinical criteria.^10-12^ Another key aspect in correctly defining survival rates in FTLD is the study design, to minimize potential biases.^13^ Indeed, many survival studies rely on cohort series, with patients recruited after disease onset; in this case, patients with faster progression and early death may fail to be recruited and are missed, in what is known as the left truncation bias.^14,15^ Conversely, survival studies that consider autopsy series, which are typically restricted to highly specialized centers, may incur the right truncation bias because patients with less aggressive disorders, who live past the end of the study, are not included.^14^ Thus, the first approach may result in estimating longer survival while the second in estimating shorter survival. Population-based registers, which consider only incident patients recruited in a well-defined geographic area and followed over time, might offer some advantages, being representative of the overall population of patients with FTLD,^13^ and might overcome some of these limitations.

Establishing survival rates, its predictors, and also potential geographic differences is critical to refining the survival model in FTLD.

Given these premises, we considered the FRONTIERS network, a multinational register established in 2018 to assess the incidence of FTLD in Europe and define the frequencies of different phenotypes in the general population.^16,17^ Taking advantage of the long-term observation of this sample of incident patients with FTLD, we set up this study, with the aim to assess (1) care home admission rate and survival rate; (2) survival rate in FTLD-associated clinical phenotypes; (3) potential differences in survival rates across Europe; and (4) predictors of survival, computing survival probability at the individual patient level.

## Methods

### Study Population

The FRONTIERS study was used in this secondary analysis of longitudinal study.

This study is based on population-based data collected in 13 centers, each with long-lasting experience in the FTLD field and with the ability to cover a well-defined geographic area, across 9 European countries (the United Kingdom, the Netherlands, Finland, Sweden, Spain, Bulgaria, Serbia, Germany, Italy). In 4 countries (the Netherlands, Finland, Germany, and Italy), the registries were split into 2 distinct administrative areas. In this work, we did not include data from Dublin, Ireland, involved in a previous study, because of administrative constraints. The FRONTIERS study design and protocol were previously published (reference 16 for further details).

All patients with a new FTLD-related diagnosis in the defined ascertainment time window and defined geographic boundaries were enrolled in the FRONTIERS study. For the purpose of this study, incident patients with FTLD retrospectively included in the registry from June 1, 2018, to May 31, 2019, and diagnosed in one of the predefined geographic areas of FRONTIERS centers were considered.

The patients included in this study met current clinical criteria for the diagnosis of syndromes associated with FTLD, namely bvFTD, semantic and nonfluent variants of PPA, FTD-MND, PSP, or CBS.^2-6,18^

FRONTIERS investigators ensured that enrolled patients fulfilled criteria for FTLD-associated syndromes,^2-6,18^ and the diagnosed were ascertained by standard procedures at each tertiary referral center, including clinical examination, standardized neuropsychological assessment at each site, and structural or functional imaging study.

Adopted inclusion criteria were the following: (1) age 18 years and older; (2) fulfilling current clinical criteria of FTLD-associated syndromes^2-6,18^; (3) diagnosis of the FTLD-related disorder made in the referral period; (4) living in the referral geographic area selected in each country for the purpose of this study; (5) having an identified informant if necessary.

Exclusion criteria were the following: other medical or psychiatric illnesses that would interfere in completing assessments/diagnosis.

Diagnosis was made by FRONTIERS investigators according to standard procedures at each tertiary referral center. An ad hoc case record form was used to collect demographic and clinical findings.

Moreover, in selected patients, CSF biomarker screening, to exclude Alzheimer disease, and/or genetic screening were performed at the discretion of the referral physician. During follow-up, the diagnosis made at the time of registration was verified and confirmed by the referral physician based on confirmed adherence to clinical criteria and FTLD-related symptoms, further ensuring patient eligibility.

The logopenic variant of PPA was not included because most of these patients have underlying Alzheimer disease pathology.^19^

By May 31, 2023, each patient was re-evaluated by in-person examination by the referral team or the patient status was ascertained through telephone call with the caregiver or by inquires to administrative electronic health records; in case of previous death of any cause, the exact date was recorded. In each FRONTIERS center, death and date of death were reported by the caregiver or by consulting national survival registers. Deaths strictly related to FTLD disorder or other causes of death (cardiovascular, respiratory, or metabolic disease, or cancer) were also recorded when available.

The study was compliant with Standards of Reporting of Neurological Disorders for observational studies.^20^

### Variables of Interest

The demographic and clinical characteristics and geographic area of residency were carefully recorded and considered in the analyses as predictors of survival.

Disease duration at diagnosis was the period between symptom onset (defined as the year in which one of the core symptoms of FTLD was first noted by the patient, family member, or health care provider or allied health professional as recorded in the medical record) and the diagnosis of an FTLD-associated syndrome.

Family history was assessed according to the modified Goldman score (GS), and for the purpose of this study, the GS was clustered into 2 subgroups: GS = 1 or 2 (where 1 is an autosomal dominant family history of FTLD or ALS and 2 is familial aggregation of 3 of more family members with dementia but not meeting criteria for 1) vs GS = 3 or 4 (where 3 is one other affected family member with dementia and 4 is no or unknown family history).^21^

Comorbidities at the time of diagnosis were recorded as follows: (1) vascular-metabolic disease (i.e., the presence of either cardiovascular disease or hypertension or hypercholesterolemia or diabetes), (2) other metabolic disorders (i.e., kidney and liver diseases), (3) autoimmune disease, (4) cancer, and (5) absence of comorbidities.

The presence or absence of extrapyramidal (i.e., bradykinesia, tremor, rigidity, dystonia, or dyskinesia) or MND (i.e., pyramidal weakness or pyramidal signs, slurred speech, or swallowing) signs/symptoms was carefully assessed. Each clinician defined whether extrapyramidal or MND signs/symptoms were sufficient to identify PSP/CBS or FTD-MND phenotypes and the possible presence/absence of additional extrapyramidal or MND signs/symptoms in other phenotypes. Disease severity at time of diagnosis was measured using the Clinical Dementia Rating (CDR) Dementia Staging Instrument plus behaviour and language domains from the National Alzheimer's Coordinating Center and FTLD modules (CDR plus NACC) rating scale Sum of Boxes (SOB).^22^

In keeping with the aim of assessing the effect of European geographic area of residency on survival rates, we classified the referral centers into 4 groups: (1) Northern Europe, that is, Finland, Sweden, and the United Kingdom; (2) Central Europe, that is, Germany and the Netherlands; (4) Eastern Europe, that is, Serbia and Bulgaria; and (4) Southern Europe, that is, Spain and Italy.

### Statistical Analysis

Continuous and categorical variables are reported as mean (standard deviation) and % (numbers), respectively. The few missing values (as reported in footnotes of Table 1) were imputed with mode (for binary variables) or mean (for continuous variables). Demographic and clinical variables were compared using the one-way analysis of variance or χ^2^ test, as appropriate.

**Table 1:** Demographic and Clinical Characteristics of Incident Patients With FTLD

Variables^a^	bvFTD (N = 107)	PPA^b^ (N = 76)	FTD-MND (N = 15)	PSP/CBS^c^ (N = 68)	Total (N = 266)	*p* Value^d^
Age at diagnosis, y	64.2 (10.1)	67.2 (7.6)	65.6 (9.2)	70.4 (7.3)	66.7 (9.0)	<0.001
Sex, female % (n)	42.1 (45)	40.8 (31)	33.3 (5)	42.6 (29)	41.4 (110)	0.92^e^
Disease duration, mo	36.9 (21.5)	33.4 (19.4)	20.9 (15.0)	33.0 (19.8)	34.0 (20.4)	0.04
Education, y	11.5 (3.6)	11.9 (3.3)	12.0 (3.7)	10.8 (3.9)	11.5 (3.6)	0.28
Family history,^f^ % (n)	37.4 (40)	34.2 (26)	13.3 (2)	25.0 (17)	32.0 (85)	0.14^e^
Extrapyramidal signs at diagnosis, % (n)	27.1 (29)	19.7 (15)	26.7 (4)	100 (68)	43.6 (116)	<0.001^e^
MND at diagnosis, % (n)	0 (0)	1.3 (1)	100 (15)	0 (0)	6.0 (16)	<0.001^e^
Vascular-metabolic comorbidities at diagnosis, % (n)	55.4 (56)	63.4 (45)	53.3 (8)	70.0 (42)	61.1 (151)	0.27^e^
CDR plus NACC at diagnosis	6.5 (4.6)	4.2 (3.6)	5.9 (4.9)	4.5 (3.9)	5.3 (4.3)	<0.001
Death, % (n)	48.6 (52)	46.1 (35)	87.7 (13)	63.2 (43)	53.8 (143)	<0.01^e^
European area, North %	29.3	16.0	10.7	44.0	100	
Central %	31.3	31.7	5.3	31.7	100	
South %	41.3	28.8	2.5	27.5	100	
East %	50.0	36.9	3.3	9.8	100	

Abbreviations: bvFTD = behavioral variant frontotemporal dementia; CBS = corticobasal syndrome; CDR plus NACC = Clinical Dementia Rating Dementia Staging Instrument plus behaviour and language domains from the National Alzheimer's Coordinating Center and frontotemporal lobar degeneration modules rating scale; FTD-MND = frontotemporal dementia with motor neuron disease; FTLD = frontotemporal lobar degeneration; MND = motor neuron disease; PPA = primary progressive aphasia; PSP = progressive supranuclear palsy.

Results are expressed as mean (SD), unless otherwise specified.

a Missing values: education (n = 23), extrapyramidal signs at diagnosis (n = 9), MND at diagnosis (n = 9), CDR plus NACC at diagnosis (n = 24), comorbidities (n = 19).

b PPA: 33 nonfluent variants of PPA; 23 semantic variants of PPA; 20 unspecified PPAs.

c PSP/CBS: 41 PSP; 27 CBS.

d One-way analysis of variance, unless otherwise specified.

e χ^2^ test.

f Positive family history of dementia, motor neuron disease, or parkinsonism in at least 1 first-degree relative as per the Goldman score = 1 or 2 (Methods for details).

Care home admission and survival were calculated as time from symptom onset or time from diagnosis to time of care home admission or death from any cause (outcome = 1) or censoring date (May 31, 2023, outcome = 0).

The Kaplan-Meier method was used for care home admission and survival analyses in different phenotypes (considering age, sex, and European geographic area of residency as covariates) and in different European geographic areas of residency (considering age, sex, and phenotypes as covariates). The Cox proportional hazards models were used to examine the effect of demographic and clinical performances on survival. The results were presented as hazard ratios (HRs) and 95% CIs, keeping in mind that the HRs were adjusted for controlling variables or other covariates, as specified in the caption of Table 2. We tested the proportional hazards assumption for Cox regression model fit plotting estimates of the time-independent coefficient betas vs time. If the proportional hazards assumption is true, each beta will be a horizontal line, that is, H0: slope = 0.^23^

**Table 2:** Cox Proportional Hazards Models of Overall Survival Considering Phenotype, European Area of Residency, and Demographic and Clinical Variables

Measure	Effect on survival from disease onset		Effect on survival from diagnosis	
	HR (95% CIs)	*p* Value	HR (95% CIs)	*p* Value
Phenotype^a^				
bvFTD vs PPA	0.92 (0.59–1.43)	0.71	0.87 (0.56–1.36)	0.55
bvFTD vs FTD-MND	5.06 (2.66–9.62)	<0.001	4.59 (2.49–8.76)	<0.001
bvFTD vs PSP/CBS	1.62 (1.03–2.54)	0.04	1.56 (1.01–2.42)	0.04
European geographic areas^b^				
Northern Europe vs Central Europe	0.81 (0.39–1.68)	0.57	0.65 (0.31–1.34)	0.24
Northern Europe vs Eastern Europe	1.53 (0.98–2.37)	0.06	1.28 (0.83–1.97)	0.26
Northern Europe vs Southern Europe	0.57 (0.36–0.91)	0.02	0.50 (0.31–0.80)	<0.01
Demographic and clinical variables^c^				
Age at diagnosis, y	—	—	1.04 (1.01–1.02)	<0.001
Age at onset, y	1.03 (1.01–1.05)	<0.001		
Sex, female	0.89 (0.63–1.25)	0.49	0.87 (0.6–1.23)	0.43
Disease duration from onset to diagnosis, y	—	—	0.67 (0.99–1.01)	0.67
Education, y	0.97 (0.92–1.03)	0.32	0.99 (0.95–1.05)	0.96
Goldman score, positive family history^d^	1.19 (0.82–1.72)	0.37	1.26 (0.87–1.84)	0.22
Comorbidities, vascular-metabolic diseases	1.09 (0.78–1.53)	0.61	1.08 (0.77–1.52)	0.64
Extrapyramidal signs at diagnosis, positive^e^	—	—	1.15 (0.82–1.62)	0.42
Motor neuron disease at diagnosis, positive	—	—	4.32 (2.35–7.94)	<0.001
CDR plus NACC at diagnosis	—	—	1.02 (0.98–1.06)	0.40
Care home admission, yes	1.18 (0.79–1.76)	0.33	1.26 (0.87–1.84)	0.22

Abbreviations: bvFTD = behavioral variant frontotemporal dementia; CBS = corticobasal syndrome; CDR plus NACC = Clinical Dementia Rating Dementia Staging Instrument plus behaviour and language domains from the National Alzheimer's Coordinating Center and frontotemporal lobar degeneration modules rating scale; FTD-MND = frontotemporal dementia with motor neuron disease; HR = hazard ratio; PPAs = primary progressive aphasias; PSP = progressive supranuclear palsy.

a Corrected for age, sex, and European geographic area (Cox regression analyses).

b Corrected for age, sex, and phenotype (Cox regression analyses).

c Corrected for European geographic area (Cox regression models).

d Positive family history of dementia, motor neuron disease, or parkinsonism in at least 1 first-degree relative, with Goldman score = 1 or 2.

e Any extrapyramidal signs/symptoms including parkinsonism, dystonia, or dyskinesia.

The survival probability score (SPS) was computed considering possible predictors. For the selection of predictors, we used the least absolute shrinkage and selection operator (LASSO) method^24^ for survival analysis. This is a penalized variable selection technique, which shrinks β-coefficients [β = ln(HR)] and produces some β-coefficients that are exactly zero. The variables whose β-coefficient is zero are then automatically deleted from the predictor set. The models were screened by tuning penalized parameters with K-fold cross-validation,^25^ with K = 10 and approximately equal-sized subsets. The nonzero β-coefficients of each predictor variable from the multivariable survival model with minimum LASSO penalty were used to generate a weighted scoring system of the predictors. An overall continuous individual SPS for each patient (i) was calculated by summing up each β-coefficient x each predictor value [s(i) = Ʃj β(j) × (ij)]. The exponential of SPS, η (i) = exp[s(i)], represents the hazard score for each patient. Higher values of η (i) correspond to a higher level of hazard and a shorter survival time based on the predictors.

We estimated the cumulative combination of each SPS predictor at 1 year (1-survival [at baseline 1-year] × exp[patient's risk score profile]) and 5 years (1-survival [at baseline 5-year] × exp[patient's risk score profile]). To assess the predictive validity of the SPS, we used the area under the receiver operating characteristic curves (AUC) and the discrimination C statistic (overall AUC) which takes into consideration the timing of events from survival data.^26^ The optimal cutoff of the SPS was defined according to the Youden index method.^27^ The cutoff was used to convert the SPS into binary data and assess the Kaplan-Meier survival curves between groups.

Two-sided values of *p* < 0.05 were considered significant. Statistical analyses were conducted with software R (version 4.3.1, R Development Core Team, 2023).

### Standard Protocol Approvals, Registrations, and Patient Consents

The central ethics committee in Lecce (N51) and the local ethics committee at each site approved the study protocol, if required. Each ethics board at each site determined whether patient consent was not required or waived.

### Data Availability

All study data, including anonymized raw and analyzed data, and materials will be available from the corresponding author on reasonable request, under the condition that the recipients do not attempt to de-anonymize data nor pass to third parties.

## Results

### Population

A total of 267 patients with FTLD-associated syndromes were included in the study. Compared with the previous study of incidence,^17^ 1 patient was excluded because diagnosis was not confirmed at follow-up. Therefore, our survival analysis was based on 266 patients.

The demographic and clinical characteristics of patients with FTLD-associated syndromes are listed in Table 1. As of the census day, 65 patients (24.4%) had been admitted to a care home during the study course and 143 patients (53.8%) had died during the 5 years of follow-up while 59 patients were still alive and at home at census day or at last ascertainment. 9 patients died directly because of COVID-19 infection (3.4%, 1 with bvFTD, 6 with PPA, 1 with FTLD-MND, and 1 with CBS) while in the other 98 cases for whom cause of death was available, we did not find any significant difference between deaths related to FTLD (41.8%) and other causes of death (58.2%) among European geographic areas (*p* = 0.126).

The median care home admission rate for FTLD was 97 months (95% CIs 86–98) from disease onset and 57 months (95% CIs 56–58) from diagnosis. Considering care home admission rates, the care home admission curves did not differ between clinical phenotypes. However, the care home admission curve was lower in Southern Europe (HR 0.42, 95% CIs 0.22–0.78, *p* < 0.01) and Eastern Europe (HR 0.25, 95% CIs 0.12–0.52, *p* < 0.001), compared with Northern Europe; no difference was found between Central Europe and Northern Europe.

### Survival in FTLD

**Figure 1:**
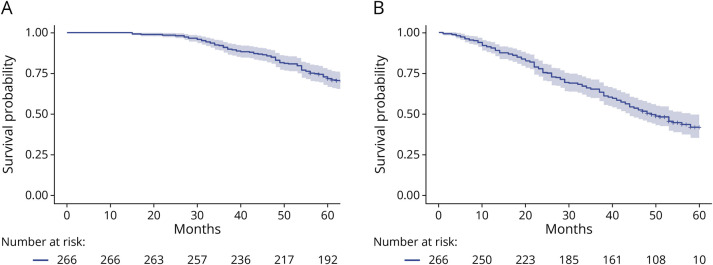
Survival Probability in Incident Patients With FTLD From Disease Onset (Panel A) and From Diagnosis (Panel B) Censored information at 60 months. FTLD = frontotemporal lobar degeneration.

The median survival for patients with FTLD-associated syndromes was 90 months (95% CIs 77–97) from onset of first symptoms (Figure 1A) and 49 months (95% CIs 44–58) from diagnosis (Figure 1B).

All fitted models met Cox proportional hazards assumption. Considering survival time from disease (and age, sex, and European geographic area as covariates), the survival curve indicated poorer prognosis in FTD-MND (HR 5.06, 95% CIs 2.66–9.62, *p* < 0.001) and PSP/CBS (HR 1.62, 95% CIs 1.03–2.54, *p* = 0.04) compared with bvFTD.

**Figure 2:**
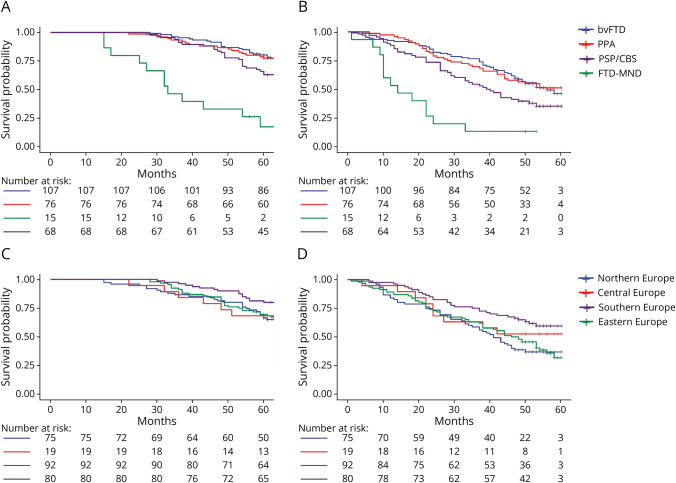
Survival Probability According to Disease Phenotypes From Disease Onset (Panel A) and From Diagnosis (Panel B) and Survival Probability in the European Area of Residency From Disease Onset (Panel C) and From Diagnosis (Panel D) Censored information at 60 months.

Regardless of time, FTD-MND showed a reduced absolute survival of 80% overall (i.e., 1–1/HR = 1–1/5) and PSP/CBS of 37% overall (1–1/1.6) compared with bvFTD. No differences in survival were found between PPA and bvFTD phenotypes (Table 2 and Figure 2A).

Comparably, considering survival time from diagnosis (and age, sex, and European geographic area as covariates), the survival curve was lower in FTD-MND (HR 4.59, 95% CIs 2.49–8.76, *p* < 0.001) and PSP/CBS (HR 1.56, 95% CIs 1.01–2.42, *p* = 0.044) compared with bvFTD; no differences were found between PPA and bvFTD phenotypes (Table 2 and Figure 2B, and eTable 1 for all group comparisons).

When we considered European geographic areas (and age, sex, and clinical phenotypes as covariates), we found significant differences in survival rates. Compared with patients living in Northern Europe, patients from Southern Europe showed better prognosis, regarding both survival time from disease onset (HR 0.57, 95% CIs 0.36–0.91, *p* = 0.02) and from diagnosis (HR 0.50, 95% CIs 031–080, *p* < 0.01). Regardless of time, patients from Northern Europe had reduced survival of 43% overall (i.e., 1-HR = 1–0.57) compared with patients from Southern Europe. No significant differences in survival curves were reported between patients living in Northern Europe and patients living in Central or Eastern Europe (Table 2 and Figure 2, C and D, and eTable 1 for all group comparisons).

Finally, considering European area of residency as strata in Cox regression analyses, age at diagnosis (HR 1.04, 95% CIs 1.01–1.02, *p* < 0.001) and MND signs at diagnosis (HR 4.32, 95% CIs 2.35–7.94, *p* < 0.001) were associated with poorer prognosis. There were no differences in hazard rates according to sex, disease duration from onset to diagnosis, education, family history (GS), or the presence of extrapyramidal symptoms at diagnosis. Cognitive impairment at the time of diagnosis, as measured by CDR plus NACC SOB, was not found to have a significant effect on survival (Table 2).

### Survival Probability Score

We used the LASSO technique for variable selection, using demographic and clinical variables as reported in Table 2 and European geographic area of residency (Southern Europe vs others). This confirmed the nonzero β-coefficients of age (β = 0.02), European geographic region (β = 0.48), and MND signs at diagnosis (β = 1.24); moreover, the LASSO technique further identified the nonzero β-coefficients of extrapyramidal symptoms at diagnosis (β = 0.008) and disease stage as measured by CDR plus NACC SOB (β = 0.0003), as independent predictors of survival rate at time of diagnosis. The SPS was generated using 4 of the 5 predictor variables reported above, as CDR plus NACC was not entered into the score as a separate variable because the β-coefficient was close to 0.

To derive the value of SPS, we considered the weighted β-coefficient scores of age (β = 0.03, HR 1.03, 95% CIs 1.01–1.06), extrapyramidal symptoms at diagnosis (β = 0.13, HR 1.14, 95% CIs 0.82–1.59), European geographic area of residency (β = 0.75, HR 2.12, 95% CIs 1.40–3.21), and MND signs at diagnosis (β = 1.15, HR 4.55, 95% CIs 2.58–8.03). The sum of the weighted scores was used to estimate the overall score.

**Figure 3:**
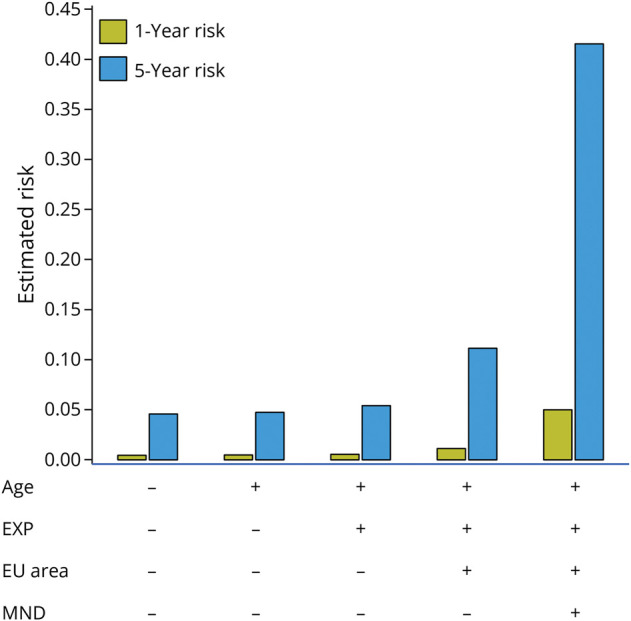
1-Year and 5-Year Estimated Risk Considering Independent Predictors Independent predictors: age (− = years at diagnosis; + = years at diagnosis plus 1); EXP = any extrapyramidal signs/symptoms including bradykinesia, rigidity, and tremor (− = absent; + = present); EU area = European geographic area of residency (− = Southern Europe; + = otherwise); MND = motor neuron disease signs at diagnosis (− = absent; + = present).

In Figure 3, we reported the estimated 1-year and 5-year risks of mortality in patients with FTLD with varied combinations of predictors: age (– = years at diagnosis; + = years at diagnosis plus 1 year); extrapyramidal symptoms at diagnosis (– = absent, + = present); European geographic area of residency (– = Southern Europe, + = others); and MND at diagnosis (– = absent, + = present). For each combination, the 5-year model gives risk estimates that are 2–3 times higher than those of the 1-year model. In particular, the 5-year estimated risk of shorter survival was clearly affected by European geographic area of residency and the presence of MND signs.

We applied a receiver operating characteristic (1-year) analysis to SPS values, observing an AUC of 0.789 (95% CIs 0.69–0.87); the optimal SPS cutoff of 2.9 (based on the Youden index) predicted 1-year survival probability with a sensitivity of 88% and a specificity of 54% (eFigure 1).

## Discussion

Survival with the syndromes associated with FTLD is highly variable, and prognostic models are essential to aid interventional trial designs and clinical management. The principal results of this multinational study are that (1) median survival is 90 months from onset of symptoms (e.g., 7.5 years) and 49 months from diagnosis (e.g., 4 years), when considering the overall FTLD spectrum; (2) there is worse prognosis in FTD-MND and PSP/CBS compared with bvFTD, but no differences between PPA and bvFTD; and (3) the SPS is able to predict prognosis at individual patient level with an accuracy of ∼80%, based on age, MND features, extrapyramidal signs, and European area of residency.

As in previous studies, we confirm that features of MND worsen the prognosis in FTLD^28-30^ and older age is an independent predictor of mortality.^31^ The survival rate also depends on the presence of extrapyramidal symptoms and European area of residency. This accords with previous work showing that development of motor impairment, irrespective of diagnostic groups and other determinants, was an adverse prognostic sign.^32^ Poorer prognosis with extrapyramidal symptoms may relate to an increased risk of complications (e.g., falls and fracture) and frailty and in turn mortality, or to a more aggressive disease associated with motor impairment.^32,33^ Our study confirms and further extends this observation, and, given the inclusion of incident patients, this work suggests how the results may be representative of the entire FTLD-associated clinical population.^13^

A novel finding was the significant difference in survival rates according to the European area of residency. We reported that patients from Southern Europe showed better prognosis, along with lower rates of care home admission, and this was independent from other variables including clinical phenotype. Again, different hypotheses might be put forward. On one hand, differences in national health system policies or different approaches to end-of-life choices may account for the different mortality rates; on the other hand, differences in FTLD-related genetic background might contribute. Indeed, in Northern Europe, and at least in Finland, the high prevalence of FTLD-associated syndromes documented by the FRONTIERS study^17^ may be related to *C9orf72* expansion founder effect, which in turn may cause a more aggressive disorder.^34^ In the same view, incidence of MND was found to vary across different ethnicities and this was associated with different rates of disease aggressiveness secondary to genetic architecture.^35-39^ It is noteworthy that life expectancy estimation of global population across European countries, with poorer prognosis in Eastern Europe and better prognosis in Northern Europe,^40^ does not match survival estimation differences in this study of patients with FTLD-associated syndromes, suggesting that our findings are disease-specific. This observation calls for reflection on the design of future pharmacologic and nonpharmacologic trials and assessment of possible confounders on treatment outcomes, considering that a rare disorder usually requires multinational cohorts. It would also be necessary to take into account and further explore differences in care home admission rates, which resulted higher in Northern Europe, to refine the survivorship model in FTLD-associated syndromes. This could be related to the spread of dementia-specific services with well-established nationwide structures in the Northern and Central Europe, or to different culture heritages with family still responsible for most of informal care in Southern and Eastern Europe.^41^

Finally, the evidence that survival of FTLD-associated syndromes is predicted by MND and extrapyramidal features calls for a transdiagnostic evaluation, not limited to the assessment of cognitive and behavioral symptoms, to better stratify patients and to tailor disease trajectory at individual patient level. The abovementioned independent variables of prognosis allowed us to compute the SPS, which proved accurate in estimating 1-year survival probability (AUC = 0.789).

The different design of this study, based on multinational population-based recruitment and current diagnostic criteria, does not allow direct comparisons with previous longitudinal or autopsy series.^10-12^ A recent population-based study, based on the Salento-Brescia register, assessed the survival rate for FTLD,^42,43^ but comparison is prevented by the exclusion of FTD-MND, the relative study homogeneity, and geographic area limited to North and South of Italy with a small source population of 2 millions of people.

We acknowledge that this study has some limitations. First, a prospective replication FRONTIERS survival study considering genetic screening is necessary to further refine and validate the prognostic model. Second, considering more detailed clinical variables, medical and psychiatric comorbidities, medications, premorbid vulnerability factors (i.e., dyslexia, autism spectrum disorder, and history of traumatic brain injury), marital status or socioeconomic status, and ethnicity could further explain some of the variations in prognosis. Third, a more detailed description of motor symptoms and their severity may help in identifying specific features of potential interest. Fourth, we cannot exclude that different evaluators, that is, neurologists, psychiatrists, or geriatricians, may have different assessment methods, and that symptom onset ascertainment, based on patient/informant reports, may be a possible source of bias. We also acknowledge that a different classification of European geographic area subgroups may influence the overall results. Finally, the generalizability of the results is prevented by lack of data from missing countries.

Despite these limitations, this study has assessed survival in FTLD-associated disorders in such a multicenter and multinational design, encompassing the wide spectrum of the diseases, revised diagnostic criteria, and incident patients.

In conclusion, the predictors and geographic differences of survival in clinical syndromes associated with FTLD may help to improve patient stratification in future clinical trials and contribute to the appropriate planning of public health service policies.

## Appendix 1 Authors

**Table:**

Name	Location	Contribution
Barbara Borroni, MD	Department of Clinical and Experimental Sciences, University of Brescia; Department of Continuity of Care and Frailty, ASST Spedali Civili, Brescia, Italy	Drafting/revision of the manuscript for content, including medical writing for content; major role in the acquisition of data; study concept or design; analysis or interpretation of data
Barbara Tarantino, PhD	Medical and Genomic Statistics Unit, Department of Brain and Behavioural Sciences, University of Pavia, Italy	Drafting/revision of the manuscript for content, including medical writing for content; analysis or interpretation of data
Caroline Graff, MD, PhD	Division of Neurogeriatrics, Department NVS, Karolinska Institutet, Solna; Unit for Hereditary Dementia, Theme Inflammation and Aging, Karolinska University Hospital-Solna, Stockholm, Sweden	Drafting/revision of the manuscript for content, including medical writing for content; major role in the acquisition of data; analysis or interpretation of data
Johanna Krüger, MD, PhD	Research Unit of Clinical Medicine, Neurology, University of Oulu; MRC, Oulu University Hospital; Neurocenter, Neurology, Oulu University Hospital, Finland	Drafting/revision of the manuscript for content, including medical writing for content; major role in the acquisition of data; analysis or interpretation of data
Albert C. Ludolph, MD	Department of Neurology, University of Ulm; Deutsches Zentrum für Neurodegenerative Erkrankungen (DZNE), Ulm, Germany	Drafting/revision of the manuscript for content, including medical writing for content; major role in the acquisition of data; analysis or interpretation of data
Fermin Moreno, MD, PhD	Cognitive Disorders Unit, Department of Neurology, Hospital Universitario Donostia; Neuroscience Area, Biogipuzkoa Health Research Institute, San Sebastian, Spain	Drafting/revision of the manuscript for content, including medical writing for content; major role in the acquisition of data; analysis or interpretation of data
Markus Otto, MD	Department of Neurology, University of Ulm; Department of Neurology, Martin Luther University, University Hospital, Halle (Saale), Germany	Drafting/revision of the manuscript for content, including medical writing for content; major role in the acquisition of data; analysis or interpretation of data
James B. Rowe, FRCP, PhD	MRC Cognition and Brain Sciences Unit, Department of Clinical Neurosciences, and Cambridge University Hospitals NHS Trust, University of Cambridge, United Kingdom	Drafting/revision of the manuscript for content, including medical writing for content; major role in the acquisition of data; analysis or interpretation of data
Harro Seelaar, MD	Department of Neurology and Alzheimer Center Erasmus MC, Erasmus MC University Medical Center, Rotterdam, the Netherlands	Drafting/revision of the manuscript for content, including medical writing for content; major role in the acquisition of data; analysis or interpretation of data
Eino Solje, MD, PhD	Neurology, Institute of Clinical Medicine, University of Eastern Finland, Kuopio; Neurocenter, Neurology, Kuopio University Hospital, Finland	Drafting/revision of the manuscript for content, including medical writing for content; major role in the acquisition of data; analysis or interpretation of data
Elka Stefanova, MD, PhD	Neurology Clinic, Faculty of Medicine, University Clinical Center, University of Belgrade, Serbia	Drafting/revision of the manuscript for content, including medical writing for content; major role in the acquisition of data; analysis or interpretation of data
Latchezar D. Traykov, MD	UH Alexandrovska, Department of Neurology, Medical University Sofia, Bulgaria	Drafting/revision of the manuscript for content, including medical writing for content; major role in the acquisition of data; analysis or interpretation of data
Vesna Jelic, MD	Theme Inflammation and Aging, Medical Unit Aging Brain, Karolinska University Hospital Huddinge, Solna; Division of Clinical Geriatrics, Department NVS, Karolinska Institutet, Huddinge, Sweden	Drafting/revision of the manuscript for content, including medical writing for content; major role in the acquisition of data; analysis or interpretation of data
Sarah Anderl-Straub, DPsych	Research Unit of Clinical Medicine, Neurology, University of Oulu, Finland	Drafting/revision of the manuscript for content, including medical writing for content; major role in the acquisition of data; analysis or interpretation of data
Anne M. Portaankorva, MD, PhD	Research Unit of Clinical Medicine, Neurology, University of Oulu; MRC, Oulu University Hospital, Finland	Drafting/revision of the manuscript for content, including medical writing for content; major role in the acquisition of data; analysis or interpretation of data
Myriam Barandiaran, MD	Cognitive Disorders Unit, Department of Neurology, Hospital Universitario Donostia; Neuroscience Area, Biogipuzkoa Health Research Institute, San Sebastian, Spain	Drafting/revision of the manuscript for content, including medical writing for content; major role in the acquisition of data; analysis or interpretation of data
Alazne Gabilondo, MD	Cognitive Disorders Unit, Department of Neurology, Hospital Universitario Donostia; Neuroscience Area, Biogipuzkoa Health Research Institute, San Sebastian, Spain	Drafting/revision of the manuscript for content, including medical writing for content; major role in the acquisition of data; analysis or interpretation of data
Alexander G. Murley, MBChB	MRC Cognition and Brain Sciences Unit, Department of Clinical Neurosciences, and Cambridge University Hospitals NHS Trust, University of Cambridge, United Kingdom	Drafting/revision of the manuscript for content, including medical writing for content; major role in the acquisition of data; analysis or interpretation of data
Timothy Rittman, BMEdSci, BMBS, PgCertMedEd, PhD, FRCP	MRC Cognition and Brain Sciences Unit, Department of Clinical Neurosciences, and Cambridge University Hospitals NHS Trust, University of Cambridge, United Kingdom	Drafting/revision of the manuscript for content, including medical writing for content; major role in the acquisition of data; analysis or interpretation of data
Emma Van Der Ende, MD	Department of Neurology and Alzheimer Center Erasmus MC, Erasmus MC University Medical Center, Rotterdam, the Netherlands	Drafting/revision of the manuscript for content, including medical writing for content; major role in the acquisition of data; analysis or interpretation of data
John C. Van Swieten, MD	Department of Neurology and Alzheimer Center Erasmus MC, Erasmus MC University Medical Center, Rotterdam, the Netherlands	Drafting/revision of the manuscript for content, including medical writing for content; major role in the acquisition of data; analysis or interpretation of data
Päivi Hartikainen, MD, PhD	Neurology, Institute of Clinical Medicine, University of Eastern Finland, Kuopio, Finland	Drafting/revision of the manuscript for content, including medical writing for content; major role in the acquisition of data; analysis or interpretation of data
Gorana Mandić Stojmenović, MD, PhD	Neurology Clinic, Faculty of Medicine, University Clinical Center, University of Belgrade, Serbia	Drafting/revision of the manuscript for content, including medical writing for content; major role in the acquisition of data; analysis or interpretation of data
Shima Mehrabian, MD	UH Alexandrovska, Department of Neurology, Medical University Sofia, Bulgaria	Drafting/revision of the manuscript for content, including medical writing for content; major role in the acquisition of data; analysis or interpretation of data
Roberta Ghidoni, PhD	Molecular Markers Laboratory, IRCCS Istituto Centro San Giovanni di Dio Fatebenefratelli, Brescia, Italy	Drafting/revision of the manuscript for content, including medical writing for content; major role in the acquisition of data; analysis or interpretation of data
Antonella C. Alberici, MD	Department of Continuity of Care and Frailty, ASST Spedali Civili, Brescia, Italy	Drafting/revision of the manuscript for content, including medical writing for content; major role in the acquisition of data; analysis or interpretation of data
Maria Teresa Dell'Abate, PhD	Center for Neurodegenerative Diseases and the Aging Brain, Pia Fondazione Cardinale Giovanni Panico, University of Bari-Aldo Moro, Italy	Drafting/revision of the manuscript for content, including medical writing for content; major role in the acquisition of data; analysis or interpretation of data
Chiara Zecca, PhD	Center for Neurodegenerative Diseases and the Aging Brain, Pia Fondazione Cardinale Giovanni Panico, University of Bari-Aldo Moro, Italy	Drafting/revision of the manuscript for content, including medical writing for content; major role in the acquisition of data; analysis or interpretation of data
Mario Grassi, PhD	Medical and Genomic Statistics Unit, Department of Brain and Behavioural Sciences, University of Pavia, Italy	Drafting/revision of the manuscript for content, including medical writing for content; analysis or interpretation of data
Giancarlo Logroscino, MD	Center for Neurodegenerative Diseases and the Aging Brain, Pia Fondazione Cardinale Giovanni Panico, University of Bari-Aldo Moro, Italy	Drafting/revision of the manuscript for content, including medical writing for content; major role in the acquisition of data; study concept or design; analysis or interpretation of data

## Appendix 2 Coinvestigators

**Table:**

Name	Location	Role	Contribution
Belezhanska Diyana, MD, PhD	UH “Alexandrovska,” Department of Neurology, Medical University Sofia, Bulgaria	Coinvestigator	Patient assessment
Bianchetti Angelo, MD	Geriatric Unit, S. Anna Hospital, Brescia, Italy	Coinvestigator	Patient assessment
Binetti Giuliano, MD	MAC Memory Clinic and Molecular Markers Laboratory, IRCCS Istituto, Centro San Giovanni di Dio Fatebenefratelli, Brescia, Italy	Coinvestigator	Patient assessment
Cotelli Maria, PhD	Neuropsychology Unit, IRCCS Istituto Centro San Giovanni di Dio Fatebenefratelli, Brescia, Italy	Coinvestigator	Patient assessment
Cotelli Maria Sofia, MD	Department of Continuity of Care and Frailty, ASST Spedali Civili, Brescia, Italy	Coinvestigator	Patient assessment
Dreharova Irena, MD	UH “Alexandrovska,” Department of Neurology, Medical University Sofia, Bulgaria	Coinvestigator	Patient assessment
Filardi Marco, MSc	Center for Neurodegenerative Diseases and the Aging Brain, Pia Fondazione Cardinale Giovanni Panico, University of Bari-Aldo Moro, Bari, Italy	Coinvestigator	Patient assessment
Fostinelli Silvia, MSc	MAC Memory Clinic and Molecular Markers Laboratory, IRCCS Istituto Centro San Giovanni di Dio Fatebenefratelli, Brescia, Italy	Coinvestigator	Patient assessment
Gnoni Valentina, MSc	Center for Neurodegenerative Diseases and the Aging Brain, Pia Fondazione Cardinale Giovanni Panico, University of Bari-Aldo Moro, Bari, Italy	Coinvestigator	Patient assessment
Haapasalo Annakaisa, PhD	A.I. Virtanen Institute for Molecular Sciences, University of Eastern Finland, Kuopio, Finland	Coinvestigator	Patient assessment
Nacheva Genoveva, MD	Institute of Molecular Biology “Roumen Tsanev,” Bulgarian Academy of Sciences, Sofia, Bulgaria	Coinvestigator	Patient assessment
Novaković Ivana, MD	Faculty of Medicine, University of Belgrade, Belgrade, Serbia	Coinvestigator	Patient assessment
Popivanov Ivo, PhD	Department for Cognitive Science and Psychology, New Bulgarian University, Sofia, Bulgaria	Coinvestigator	Patient assessment
Raycheva Margarita, MD	UH “Alexandrovska,” Department of Neurology, Medical University Sofia, Bulgaria	Coinvestigator	Patient assessment
Rivolta Jasmine, MSc	Department of Continuity of Care and Frailty, ASST Spedali Civili, Brescia, Italy	Coinvestigator	Patient assessment
Stockton Katherine, MD	Department of Clinical Neurosciences, MRC Cognition and Brain Sciences Unit, and Cambridge University Hospitals NHS Trust, University of Cambridge, United Kingdom	Coinvestigator	Patient assessment
Stoyanova Katya, MD	UH “Alexandrovska,” Department of Neurology, Medical University Sofia, Bulgaria	Coinvestigator	Patient assessment
Suhonen Noora-Maria, MSc	Research Unit of Clinical Medicine, Neurology, University of Oulu, Finland and Neurocenter, Neurology, Oulu University Hospital, Finland	Coinvestigator	Patient assessment
Taheri Rydell Melissa, MD	Department NVS, Division of Neurogeriatrics, Karolinska Institutet, Solna, Sweden; and Unit for Hereditary Dementia, Theme Inflammaion and Aging, Karolinska University Hospital-Solna, Stockholm, Sweden	Coinvestigator	Patient assessment
Tainta Mikel, MD	Neuroscience Area, Biogipuzkoa Health Research Institute, San Sebastian, Spain	Coinvestigator	Patient assessment
Toncheva Draga, MD	Department of Genetics, Medical University Sofia, Bulgaria	Coinvestigator	Patient assessment
Urso Daniele, MD	Center for Neurodegenerative Diseases and the Aging Brain, Pia Fondazione Cardinale Giovanni Panico, University of Bari-Aldo Moro, Bari, Italy	Coinvestigator	Patient assessment
Zlatareva Dora, MD	UH “Alexandrovska,” Department of Radiology, Medical University Sofia, Sofia, Bulgaria	Coinvestigator	Patient assessment

## Glossary

AUC: area under the receiver operating characteristic curve
bvFTD: behavioral variant FTD
CBS: corticobasal syndrome
CDR: Clinical Dementia Rating
FTLD: frontotemporal lobar degeneration
FTD: frontotemporal dementia
FTD-MND: FTD with motor neuron disease
GS: Goldman score
LASSO: least absolute shrinkage and selection operator
NACC: National Alzheimer's Coordinating Center
PPA: primary progressive aphasia
PSP: progressive supranuclear palsy
SOB: Sum of Boxes
SPS: survival probability score
